# Oritavancin for Gram-Positive Bone and Joint Infections: A Comprehensive Review of the Literature

**DOI:** 10.3390/antibiotics15020226

**Published:** 2026-02-19

**Authors:** Zain Ahmed Raza, Alex Giannini, Marco Bongiovanni

**Affiliations:** 1 Division of Infectious Diseases, Ente Ospedaliero Cantonale, 6900 Lugano, Switzerland; 2 Department of Biomedical Sciences, University of Southern Switzerland, 6900 Lugano, Switzerland

**Keywords:** oritavancin, long-acting lipoglycopeptide, osteomyelitis, bone, joint, infection Bacteremia

## Abstract

**Background:** Bone and joint infections (BJIs), including osteomyelitis, septic arthritis, and periprosthetic joint infections, typically require prolonged antimicrobial therapy and often involve complex outpatient management. Oritavancin, a long-acting lipoglycopeptide approved for the treatment of acute bacterial skin and skin structure infections caused by Gram-positive bacteria, has emerged as a potential off-label option for BJIs owing to its favourable pharmacokinetic and pharmacodynamic properties. **Objectives:** To provide a comprehensive overview of the pharmacological rationale, microbiological activity, and available clinical evidence supporting the use of oritavancin in BJIs. **Methods:** A comprehensive narrative review of the literature was performed using MEDLINE and the Cochrane Central Register of Controlled Trials (CENTRAL), focusing on publications from 2011 to 2025. Observational studies, case series, and case reports describing the off-label use of oritavancin in BJIs were considered. **Results:** The available literature primarily consists of observational studies and real-world experiences. Eighteen studies met the inclusion criteria. Oritavancin was most frequently evaluated for osteomyelitis (n = 14 studies), prosthetic joint infections (n = 10) and septic arthritis (n = 5). Multi-dose regimens, typically including a 1200 mg loading dose followed by weekly doses of 800–1200 mg, were the most commonly described strategies. Reported clinical success rates generally ranged from approximately 70% to over 90%. Oritavancin was overall well tolerated, with adverse events being mostly mild and self-limiting. **Conclusions:** Current evidence suggests that oritavancin may represent an effective and well-tolerated off-label option for selected patients with Gram-positive BJIs. Its use may offer practical advantages, including reduced hospitalization and avoidance of prolonged intravenous antimicrobial therapy, particularly in patients for whom standard treatment approaches are challenging.

## 1. Introduction

Bone and joint infections (BJIs) remain a major clinical challenge, often requiring prolonged intravenous (IV) antimicrobial therapy and coordinated multidisciplinary management [1,2]. For osteomyelitis, current guidelines generally recommend at least six weeks of antimicrobial treatment following adequate surgical debridement, although longer courses may be needed in the presence of multidrug-resistant pathogens, atypical organisms, inadequate source control, or persistent radiological or clinical signs of infection. Similarly, in prosthetic joint infections (PJIs) among patients who are not surgical candidates, guidelines suggest 4–6 weeks of pathogen-directed IV or highly bioavailable oral therapy, often followed by indefinite suppressive antimicrobial treatment [3,4].

Extended antibiotic courses are frequently associated with prolonged hospitalization, increased healthcare costs, cumulative drug toxicities, and a higher risk of antimicrobial resistance. Moreover, long-term IV therapy may negatively impact patients’ quality of life and adherence, particularly in frail individuals or those with multiple comorbidities. These challenges underscore the need for alternative therapeutic strategies, especially against methicillin-resistant *Staphylococcus aureus* (MRSA) and vancomycin-resistant enterococci (VRE), which remain among the most difficult-to-treat pathogens in BJIs and are associated with poor clinical outcomes [5].

Conventional IV regimens pose additional challenges for outpatient parenteral antimicrobial therapy (OPAT) programs, including catheter-related complications, bloodstream infections, thrombosis, and logistical barriers to frequent healthcare access. Vulnerable populations, such as people who inject drugs, are at particularly high risk of treatment failure or discontinuation [5,6]. From a healthcare perspective, prolonged hospitalization and OPAT programs also represent a substantial economic burden [7].

Treatment failures in bone and joint infections carry substantial clinical and societal consequences. Persistent infection can lead to chronic pain, functional impairment, and, in severe cases, limb loss, profoundly affecting patient quality of life. Recurrent or inadequately treated infections may necessitate additional surgeries, prolonged rehabilitation, and repeated hospitalizations, increasing healthcare resource utilization. Moreover, suboptimal antimicrobial therapy contributes to the emergence of resistant strains, amplifying both individual and public health risks. These considerations underscore the importance of effective, patient-centred treatment strategies that balance clinical efficacy, safety, practicality and feasibility in real-word clinical settings.

BJIs are associated with significant morbidity and measurable mortality, particularly among older patients and those with multiple comorbidities. Recent studies report short- and mid-term mortality rates of approximately 5–15% in PJIs and even higher rates in vertebral osteomyelitis, with up to 15–20% mortality within the first year after diagnosis [8,9]. Mortality is influenced by host factors, delayed diagnosis, inadequate source control, and infections caused by resistant Gram-positive pathogens, including MRSA and VRE [10]. These data highlight the need for effective and durable antimicrobial strategies that ensure sustained pathogen suppression while minimizing treatment-related complications, particularly in frail or high-risk patients.

Oritavancin, a long-acting lipoglycopeptide antibiotic, has emerged as a promising option for Gram-positive infections [11,12,13]. Its triple mechanism of action confers potent bactericidal activity, including against strains with reduced susceptibility to other glycopeptides. Thanks to its exceptionally long terminal half-life, oritavancin can be administered as a single dose or in a limited number of infusions, potentially minimizing the need for long-term IV access and facilitating early hospital discharge or fully outpatient management [14]. Furthermore, oritavancin demonstrates broad-spectrum activity against clinically relevant Gram-positive pathogens, including MRSA and VRE, offering an attractive alternative to standard agents such as vancomycin, daptomycin, or linezolid [15,16,17,18,19,20,21].

Although oritavancin is currently approved by the U.S. Food and Drug Administration for acute bacterial skin and skin structure infections (ABSSSI), a growing body of observational studies and real-world experiences suggests its potential utility in more complex, deep-seated infections, including osteomyelitis, septic arthritis, and infective endocarditis [14,20,22,23,24,25]. This off-label use is pharmacologically plausible given oritavancin’s sustained bactericidal activity against non-replicating bacteria, effectiveness against biofilm-embedded microorganisms, and favourable bone penetration demonstrated in preclinical models [26].

In light of the expanding but heterogeneous literature, this comprehensive review provides an integrated overview of oritavancin in Gram-positive BJIs. By critically summarizing case reports, observational studies, and narrative syntheses published between 2011 and 2025, this review evaluates clinical outcomes, safety, and practical considerations, offering a clinically relevant perspective for infectious disease specialists and orthopaedic teams.

## 2. Literature Overview and Data Sources

The literature summarized in this review was selected to provide a broad, clinically meaningful overview of oritavancin use in BJIs. Unlike a systematic review, which relies on rigid methodological criteria, this comprehensive narrative approach aims to capture the full spectrum of available clinical experience, including larger observational cohorts as well as smaller case reports that may offer insights into rare or complex scenarios.

Relevant publications were identified through searches of MEDLINE/PubMed, the Cochrane Central Register of Controlled Trials (CENTRAL), and Google Scholar, focusing on articles published between January 2011 and March 2025. The search strategy combined Medical Subject Headings (MeSH) terms and free-text keywords as follows: (Oritavancin or oritavancin) and (“Bone and Bones” or “Orthopedic Procedures” or osteomyelitis or “bone infection*” or “orthopedic infection*” or “prosthetic joint infection*” or “periprosthetic joint infection*” or “joint infection*” or osteitis or “septic arthritis”. To ensure completeness, reference lists of key articles were also manually screened for additional relevant reports. Inclusion criteria comprised studies enrolling adult patients (≥18 years) with a diagnosis of BJI suspected or microbiologically confirmed to be caused by Gram-positive pathogens and treated with oritavancin as part of the antibiotic regimen. Exclusion criteria included studies not written in English, pediatric studies, in vitro or animal studies, pharmacokinetic-only analyses, conference abstracts, secondary analyses of previously published data, and studies lacking information on clinical efficacy and/or safety outcomes.

Given the absence of randomized controlled trials specifically evaluating oritavancin for BJIs, the review prioritized real-world clinical evidence. This included retrospective cohort studies, case–control studies, case series, and individual case reports involving adult patients with suspected or microbiologically confirmed Gram-positive BJIs treated with oritavancin [27,28,29,30,31,32,33,34,35,36,37,38,39,40,41,42,43]. Narrative reviews and large observational programs were also included when they provided relevant safety data or contextual information. No restrictions were applied regarding infection chronicity, prior antimicrobial exposure, or concomitant surgical interventions, reflecting the heterogeneity of real-world practice.

The selected studies were evaluated qualitatively, with the aim of identifying common clinical patterns, practical dosing strategies, safety signals, and reported outcomes. Rather than applying formal meta-analytic techniques, key elements—such as infection type, microbiological etiology, oritavancin regimen, treatment setting (inpatient versus outpatient), follow-up duration, and clinical outcomes—were summarized descriptively and integrated into a narrative discussion.

A total of 83 studies evaluating the use of oritavancin in BJIs were initially identified (57 from PubMed, 6 from CENTRAL, and 20 from Google Scholar). After removal of duplicates (n = 16), 49 studies were excluded for the following reasons: non-clinical research, in vitro or animal studies, lack of efficacy or safety data, pharmacokinetic analyses only, conference papers, or failure to meet inclusion criteria. Finally, 18 studies were included in the final review.

A formal risk-of-bias assessment was not conducted, as the majority of studies were observational and highly heterogeneous. Instead, the review emphasizes consistency across studies, biological plausibility of observed outcomes, and the alignment of findings with known pharmacokinetic and pharmacodynamic properties of oritavancin.

This approach aligns with the objectives of a comprehensive review: to synthesize diverse sources of evidence, highlight areas of consensus and uncertainty, and provide clinically relevant guidance for practitioners. By integrating data from a variety of study designs, this section offers a balanced perspective on current knowledge while acknowledging the inherent limitations of the available literature.

## 3. Pharmacological Rationale for Use in Bone and Joint Infections

Oritavancin exhibits potent antimicrobial activity against a broad spectrum of Gram-positive aerobic bacteria, including enterococci (notably vancomycin-resistant enterococci), staphylococci (both methicillin-susceptible and methicillin-resistant *S. aureus*), and streptococci such as *S. pyogenes*, *S. agalactiae*, *S. dysgalactiae*, and members of *S. anginosus* group. In addition, it demonstrates activity against several anaerobic Gram-positive organisms, including *Clostridium difficile*, *Clostridium perfringens*, *Peptostreptococcus* spp., and *Propionibacterium acnes*. In vitro activity has also been described against *Micrococcus* spp., *Corynebacterium* spp., and *Listeria monocytogenes* [44,45,46].

**Figure 1:**
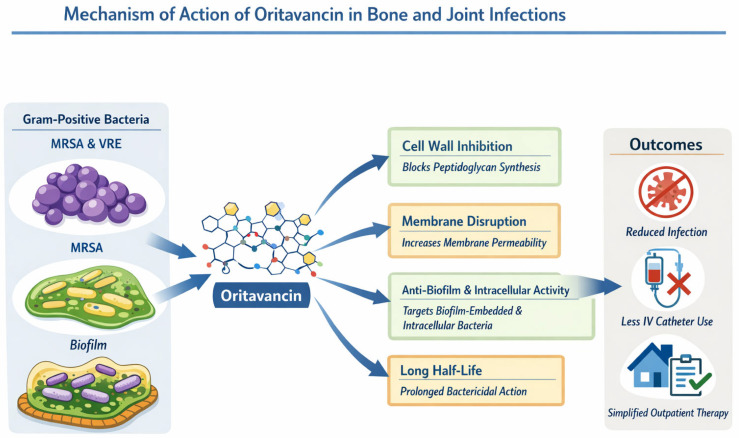
Mechanism of action of oritavancin in bone and joint infections. MRSA: methicillin-resistant *Staphylococcus aureus*; VRE: vancomycin-resistant enterococci; IV: intravenous.

Oritavancin is characterized by a prolonged terminal half-life of approximately 245 h and concentration-dependent bactericidal activity, best correlated with the maximum plasma concentration to MIC ratio (C_max/MIC). Owing to its long half-life, additional pharmacodynamic parameters such as time above MIC (T > MIC) and area under the concentration–time curve to MIC ratio (AUC/MIC) also contribute to its antibacterial efficacy. Preclinical animal studies have demonstrated that oritavancin achieves bone concentrations exceeding those observed in plasma, with bone AUC values up to threefold higher than serum levels. Importantly, drug concentrations remained above the MIC_90 for *S. aureus* throughout the study period, supporting its potential role in the treatment of osteomyelitis [47]. A schematic illustration of oritavancin’s mechanisms of action in bone and joint infections, including its activity against Gram-positive bacteria, biofilm-embedded and intracellular pathogens, and its sustained bactericidal effect, is shown in Figure 1.

Furthermore, in vitro studies have shown that oritavancin has superior intracellular activity against *S. aureus* compared with vancomycin, achieving reductions of 3–4 log units in intracellular bacterial counts. Oritavancin has also demonstrated significant anti-biofilm activity against staphylococci, including MRSA and *S. epidermidis*, with reported synergistic effects when combined with rifampin or gentamicin, a feature particularly relevant for biofilm-associated BJIs [48].

## 4. Clinical Evidence in Bone and Joint Infections

The clinical evidence for oritavancin in BJIs is almost entirely derived from observational studies, real-world experiences, case series, and single case reports (Table 1). While these sources provide valuable insights into practical feasibility, dosing strategies, and potential effectiveness, they are inherently limited by study design, heterogeneity of patient populations, infection severity, prior therapies, and outcome definitions. Therefore, the current evidence should be interpreted as hypothesis-generating rather than confirmatory.

Osteomyelitis represents the most frequently reported indication for oritavancin use. MRSA is the predominant pathogen, reflecting both its prevalence in BJIs and the clinical challenges posed by resistant strains. Retrospective cohort studies suggest that weekly or multi-dose regimens of oritavancin can achieve favourable outcomes, with clinical success rates commonly reported between 70% and 80% [28,34,36]. In the largest multicenter retrospective analysis, oritavancin-treated patients demonstrated higher rates of clinical resolution, fewer readmissions, and reduced need for repeat surgical interventions compared with daptomycin [34]. While encouraging, these findings must be interpreted with caution, as baseline differences between patient groups and potential confounding by indication limit the ability to attribute improved outcomes solely to oritavancin.

Comparative evidence with other long-acting lipoglycopeptides, such as dalbavancin, suggests broadly similar success rates in osteomyelitis (70–85%) [33,38]. However, differences in dosing schedules, prior antibiotic exposure, concomitant surgical procedures, and definitions of clinical success complicate direct comparisons. This underscores the need for careful interpretation and highlights the challenges of synthesizing evidence across heterogeneous real-world cohorts.

Real-world outpatient studies provide particularly useful insights into the feasibility of oritavancin for patients in whom daily IV therapy is impractical. Across multiple infusion centers, patients with high rates of MRSA infection and prior treatment failures achieved clinical success in approximately 75–80% of cases [28,36]. These studies also reported low rates of adverse events and minimal need for hospitalization, highlighting the potential of oritavancin to simplify care and reduce healthcare resource utilization. Nonetheless, follow-up durations were typically limited to 3–6 months, leaving the long-term durability of response, relapse rates, and functional recovery largely uncharacterized.

Evidence for prosthetic joint infections (PJIs) and septic arthritis is more limited and less consistent. Success rates hover around 70% in small cohorts and registry data [29,38,49], with failures often observed in patients receiving single-dose regimens for active infections. These findings suggest that insufficient antimicrobial exposure may contribute to suboptimal outcomes in biofilm-associated infections, where bacteria are shielded from host defences and antibiotics. Surgical management variability, prior antibiotic exposure, and the frequent use of oritavancin as salvage or consolidation therapy further complicate interpretation.

Case reports and small series offer additional perspectives on complex scenarios, including chronic osteomyelitis, vertebral infections, and VRE infections [37,41,42]. In these reports, oritavancin was often administered after failure or intolerance of standard therapies and generally led to favorable clinical outcomes. While these individual experiences are encouraging, they are inherently prone to publication bias, as unsuccessful cases are less likely to be reported.

Several factors appear to influence clinical outcomes with oritavancin in BJIs. Adequate surgical debridement, pathogen susceptibility, biofilm presence, and host comorbidities such as diabetes or immunosuppression are important determinants of success. Observational data suggest that multi-dose regimens are more likely to succeed in patients with complex infections, particularly when combined with appropriate surgical intervention. Understanding these predictive factors may aid in patient selection and optimize therapeutic strategies in real-world practice.

An emerging area of interest is the use of therapeutic drug monitoring (TDM) to guide individualized oritavancin therapy. Selected case reports have demonstrated sustained plasma concentrations above pathogen-specific MICs, supporting pharmacologically rational dosing [23,27,50,51]. Despite the mechanistic plausibility, TDM remains anecdotal, with no validated PK/PD targets or established clinical guidelines. Its routine implementation is currently limited and requires further investigation to determine whether it improves outcomes or safety.

Large observational programs, such as the CHROME study, provide broader insights into real world practice [49]. Among patients treated for osteomyelitis or joint infections, high rates of clinical success were generally observed, particularly with multi-dose regimens. However, the retrospective design, heterogeneous infection types, and lack of standardized outcome definitions prevent definitive conclusions regarding comparative efficacy.

In summary, the current body of evidence suggests that oritavancin may be a valuable therapeutic option for selected Gram-positive BJIs, particularly osteomyelitis, where prolonged antimicrobial exposure is needed and standard IV regimens are impractical. However, the predominance of observational data, heterogeneous dosing strategies, and limited long-term follow-up underscore the need for cautious interpretation. High-quality prospective studies are required to define optimal patient selection, dosing regimens, and the comparative effectiveness of oritavancin relative to standard-of-care therapies.

## 5. Safety and Tolerability

Across the studies, oritavancin demonstrated a generally favorable safety and tolerability profile, even when administered in prolonged or multiple dose regimens. Reported adverse events were infrequent and predominantly mild to moderate. The most common events included nausea, headache, diarrhea, pruritus, and skin rash, all of which were typically self-limiting and did not require treatment discontinuation [52].

Infusion-related reactions were only occasionally reported, consistent with the known pharmacological properties of glycopeptide antibiotics. Due to the prolonged terminal half-life of oritavancin, these reactions may persist longer than with shorter-acting agents; however, they were generally manageable with infusion rate adjustment or symptomatic treatment. Importantly, no severe infusion-related hypersensitivity reactions were observed across studies. Transient laboratory abnormalities were reported in a limited number of cases, as mild and reversible elevations in liver transaminases, without evidence of clinical hepatotoxicity [51]. Notably, no consistent signals of cumulative toxicity, nephrotoxicity, hematologic abnormalities, or cardiac adverse events were identified, even among patients receiving repeated weekly doses over several weeks. Overall, tolerability of oritavancin in real world BJI treatment appeared comparable to, or in some cases more favorable than, standard-of-care intravenous therapies requiring daily administration. The absence of a need for prolonged central venous access may further reduce the risk of catheter-related complications, indirectly contributing to an improved safety profile. Taken together, available data support the feasibility of oritavancin use in outpatient parenteral antimicrobial therapy (OPAT) programs and in patients for whom long-term intravenous access is undesirable or impractical.

## 6. Dosing Strategies

Most successful regimens involved a 1200 mg loading dose followed by weekly or longer-term 800–1200 mg doses for 4–6 administrations. Some reports describe successful outcomes with single-dose regimens, though multiple dose strategies appear more reliable for deep-seated infections. TDM has been proposed in prolonged or suppressive regimens, aiming for trough concentrations >3 mg/L [52] and adequate AUC/MIC ratios [23,27,51,52].

## 7. Discussion

BJIs are inherently complex conditions that frequently require prolonged antimicrobial therapy, repeated surgical interventions, and close multidisciplinary management. The challenges of treating BJIs are compounded by the presence of resistant organisms, biofilm formation, and patients with multiple comorbidities. In this context, long-acting lipoglycopeptides such as oritavancin have emerged as attractive alternatives, offering the possibility of sustained antimicrobial exposure with simplified dosing schedules.

The evidence summarized in this review suggests that oritavancin can achieve high rates of clinical success, particularly in osteomyelitis, where sustained drug exposure is critical for eradicating slow-growing or biofilm-associated Gram-positive bacteria. Multi-dose regimens appear to be more reliable than single-dose strategies, likely because repeated exposure ensures maintenance of therapeutic drug concentrations in bone and joint tissues over time. Failures reported in single-dose regimens highlight the risk of under treatment in deep-seated infections and suggest that dosing strategy should be carefully tailored to infection complexity and microbial burden.

Oritavancin’s pharmacokinetic and pharmacodynamic properties provide a compelling mechanistic rationale for its use in BJIs. Its prolonged half-life allows for weekly or extended-interval dosing, maintaining effective concentrations at the site of infection, including bone and intracellular compartments. Its broad-spectrum activity against MRSA, VRE, and other clinically relevant Gram-positive pathogens, coupled with demonstrated anti-biofilm properties, further supports its potential in complex scenarios such as prosthetic joint infections.

Beyond antimicrobial efficacy, oritavancin offers significant practical advantages. Reduced need for prolonged intravenous access and feasibility of outpatient administration can improve patient adherence, minimize catheter-related complications, and reduce healthcare resource utilization. Real-world studies report lower readmission rates, fewer repeat surgical interventions, and reduced hospitalization costs, highlighting potential system-level benefits. These considerations are particularly relevant in frail or comorbid populations and in healthcare settings aiming to optimize OPAT programs and hospital throughput. Moreover, simplified dosing regimens may improve patient quality of life, allowing greater mobility, fewer hospital visits, and reduced disruption to daily activities, which are often major concerns in long-term BJI management.

Beyond efficacy, safety, and practical advantages, mortality represents a critical outcome in BJIs that reflects the combined impact of infection severity, host factors, and treatment adequacy. While BJIs are often primarily associated with morbidity, evidence indicates substantial excess mortality in high-risk populations, including older adults, immunocompromised patients, and those with multiple comorbidities. Delayed diagnosis, inadequate surgical source control, virulent or resistant Gram-positive pathogens, and treatment failure are key contributors to adverse outcomes. Recurrent infection and prolonged hospitalization further increase the risk of clinical deterioration, functional decline, and systemic complications.

Although current studies do not allow definitive conclusions regarding direct effects of specific antimicrobials on survival, long-acting agents such as oritavancin may indirectly mitigate mortality-related risks. Its prolonged bactericidal activity, limited intravenous access requirements, and feasibility for outpatient administration can reduce catheter-related complications, improve adherence, and limit the consequences of recurrent infection. By addressing key drivers of treatment failure, particularly biofilm-associated infections and resistant organisms, oritavancin has the potential to influence meaningful clinical endpoints beyond microbiological cure.

These considerations underscore the importance of including mortality and long-term survival as predefined outcomes in future research. Prospective studies comparing conventional intravenous therapy with long-acting lipoglycopeptides, alongside measures of functional recovery and quality of life, are needed to establish whether the pharmacological and logistical advantages of oritavancin translate into improved patient-centered outcomes.

In complex BJIs, particularly those involving prosthetic material or extensive biofilm formation, monotherapy may not always achieve optimal outcomes. Adjunctive or combination therapy has been explored to enhance bactericidal activity, improve biofilm penetration, and reduce the risk of treatment failure or resistance. In vitro and clinical studies suggest that oritavancin can act synergistically with agents such as rifampin or gentamicin against biofilm-embedded *S. aureus* and coagulase-negative staphylococci. This approach may be especially relevant in multi-drug-resistant infections or in patients with limited surgical options, where achieving sustained bacterial eradication is critical. While evidence remains limited to case reports and small series, combination therapy represents a rational strategy to optimize outcomes in high-risk BJIs and warrants further prospective investigation.

Nevertheless, several clinical challenges remain. Infections associated with prosthetic devices or extensive biofilm present persistent difficulties, as achieving adequate drug penetration and maintaining bactericidal concentrations over prolonged periods are critical for eradication. Similarly, immunocompromised patients, those with impaired vascular supply, or individuals with multiple prior antibiotic exposures may require tailored regimens, careful monitoring, and potentially combination therapy to ensure optimal outcomes. The variability in dosing schedules reported across studies further complicates clinical decision-making, underscoring the need for individualized strategies based on infection severity, microbial burden, and patient-specific pharmacokinetic considerations.

TDM represents an emerging but underexplored strategy for optimizing oritavancin therapy. Preliminary reports suggest that TDM may help maintain drug exposure above pathogen-specific MICs, particularly in patients with altered pharmacokinetics or extensive disease. Nonetheless, validated PK/PD targets are lacking, and the clinical utility, cost-effectiveness, and feasibility of TDM in routine practice remain uncertain. Further prospective investigation is warranted to clarify whether TDM-guided strategies can improve outcomes or reduce the risk of toxicity in complex BJIs.

Finally, integrating oritavancin into a broader patient-centred and healthcare systems perspective highlights potential benefits beyond antimicrobial efficacy. Reduced hospitalization, fewer clinic visits, and decreased reliance on long-term IV catheters may contribute to lower healthcare costs, more efficient use of inpatient resources, and improved patient satisfaction. These system-level advantages are particularly relevant in resource-limited settings or in populations facing social and logistical barriers to prolonged therapy. However, careful selection of patients is essential, as the success of oritavancin-based regimens depends on appropriate infection type, surgical management, and multidisciplinary coordination.

In conclusion, available evidence positions oritavancin as a promising option for selected patients with Gram-positive BJIs, particularly those for whom standard intravenous therapy is impractical or poorly tolerated. Its pharmacological properties, observed clinical effectiveness, and logistical advantages support consideration within individualized, multidisciplinary treatment plans. Nonetheless, well-designed prospective trials are needed to refine dosing strategies, define optimal patient selection, and assess long-term outcomes to fully establish its role in contemporary BJI management.

## 8. Limitations

The comprehensive review of oritavancin in BJIs must be interpreted with several important caveats. First, the evidence base is predominantly observational, consisting of retrospective cohorts, case series, and single case reports. The lack of randomized controlled trials precludes definitive conclusions about efficacy, comparative effectiveness, or optimal treatment strategies.

Second, there is considerable heterogeneity across studies. Infection types ranged from acute to chronic osteomyelitis, prosthetic joint infections, septic arthritis, and vertebral infections, often with variable degrees of surgical intervention and source control. Microbiological etiology was dominated by MRSA, limiting generalizability to infections caused by other Gram-positive pathogens. Patient populations were also diverse, including differences in age, comorbidities, prior antimicrobial exposure, and baseline functional status.

Third, dosing strategies for oritavancin were highly variable. Reported regimens included single doses, weekly multi-dose schedules, and extended or suppressive courses, often empirically determined rather than based on validated PK/PD targets. The absence of standardized dosing guidelines introduces uncertainty regarding optimal treatment duration, dose selection, and timing of re-administration.

Fourth, outcome definitions were inconsistent, with many studies relying on pragmatic measures of clinical improvement rather than standardized or validated criteria. Follow-up durations were frequently limited, and long-term outcomes such as relapse rates, functional recovery, and quality of life were rarely reported. These gaps limit the ability to assess the durability of response and the true effectiveness of therapy over time.

Publication bias is also likely, as positive outcomes and successful salvage cases are more frequently reported than failures. This may result in overestimation of clinical success rates. Furthermore, most studies did not include rigorous comparator groups, making it difficult to assess relative efficacy versus standard-of-care therapies or other long-acting lipoglycopeptides.

Finally, while therapeutic drug monitoring has been proposed as a potential tool to optimize therapy, the evidence remains anecdotal. The absence of validated targets, limited availability of assays, and lack of standardized protocols restrict its broader applicability.

Overall, these limitations highlight the need for cautious interpretation of the current literature and underscore the importance of well-designed prospective studies to define the optimal role of oritavancin in BJIs.

## 9. Future Perspectives

The growing body of evidence supporting the off-label use of oritavancin in BJIs highlights several important directions for future research and clinical implementation. While current data suggest that oritavancin may offer a valuable alternative to standard-of-care therapies, particularly in complex or difficult-to-treat infections, significant knowledge gaps remain that warrant further investigation.

First, well-designed prospective randomized controlled trials are urgently needed to better define the role of oritavancin in the management of BJIs. Future studies should aim to compare oritavancin-based regimens with established standard therapies, including both conventional intravenous antibiotics and other long-acting lipoglycopeptides, using standardized definitions of clinical cure and long-term follow-up. These trials would help clarify not only efficacy and safety, but also patient selection criteria, optimal timing of therapy initiation, and appropriate endpoints for treatment success.

Second, optimization of dosing strategies represents a critical area for future research. Current off-label use of oritavancin is characterized by substantial heterogeneity in dosing regimens, including variations in loading doses, maintenance doses, dosing intervals, and total treatment duration. Prospective pharmacokinetic and pharmacodynamic studies are needed to identify meaningful exposure targets associated with microbiological eradication and clinical cure in deep-seated infections such as BJIs. Establishing validated PK/PD targets could facilitate evidence-based dosing algorithms and reduce reliance on empiric or arbitrary re-dosing schedules.

In this context, the role of TDM deserves further exploration. Preliminary clinical experiences suggest that TDM-guided dosing may help ensure sustained drug exposure above pathogen-specific MICs, particularly in patients with extensive disease burden, altered pharmacokinetics, or prolonged suppressive therapy. Future studies should aim to determine clinically relevant plasma concentration thresholds, assess the feasibility of routine TDM in clinical practice, and evaluate whether TDM-guided strategies translate into improved outcomes or reduced toxicity.

Another promising area of investigation involves the use of oritavancin in combination therapy. Given its potent activity against Gram-positive pathogens, intracellular persistence, and anti-biofilm properties, oritavancin may act synergistically with other agents such as rifampin or gentamicin, particularly in biofilm-associated infections and prosthetic joint infections. Controlled studies assessing combination regimens could better define the scenarios in which adjunctive therapy provides added benefit, while minimizing the risk of resistance development or adverse effects.

Future research should also focus on expanding real-world evidence through large, multicenter registries and pragmatic observational studies that include diverse patient populations often under-represented in clinical trials, as individuals with substance use disorders, limited access to healthcare, or significant comorbidities. These data would be particularly valuable in assessing long-term outcomes, relapse rates, and functional recovery, as well as identifying predictors of treatment success or failure.

From a health systems perspective, further evaluation of the economic and logistical impact of oritavancin is warranted. Formal cost-effectiveness analyses incorporating drug acquisition costs, hospitalization duration, readmission rates, and outpatient management expenses could provide a more comprehensive understanding of the value of oritavancin-based strategies. These analyses may be especially relevant for healthcare systems seeking to reduce inpatient utilization and expand OPAT programs.

Finally, future perspectives should include the potential integration of oritavancin into antimicrobial stewardship frameworks. Clear guidance on appropriate indications, dosing regimens, and monitoring strategies could help ensure judicious use and prevent inappropriate overextension of off-label therapy. Development of consensus recommendations or expert guidelines, informed by emerging evidence, would support standardized and responsible clinical application.

In conclusion, oritavancin holds considerable promise as a therapeutic option for BJIs beyond its current approved indications. Advancing its role in clinical practice will require coordinated efforts in clinical research, pharmacological optimization, health economics, and stewardship. Addressing these future directions may ultimately contribute to improved outcomes, greater treatment flexibility, and more patient-centred care in the management of complex BJIs.

## 10. Conclusions

Bone and joint infections remain among the most challenging infections in clinical practice, often requiring prolonged antimicrobial therapy, careful surgical management, and close multidisciplinary coordination. Current evidence, although largely derived from observational studies, indicates that oritavancin may offer a viable therapeutic option for selected Gram-positive infections, particularly osteomyelitis. Its prolonged half-life and sustained antimicrobial activity enable simplified dosing schedules, which can reduce hospitalization, minimize the need for long-term intravenous access, and facilitate outpatient management.

Clinical outcomes reported across diverse patient populations suggest that multi-dose oritavancin regimens are generally effective and well tolerated, even in cases involving resistant pathogens such as MRSA and VRE. Moreover, the potential logistical and economic advantages, including fewer readmissions and reduced treatment complexity, highlight its value not only at the individual patient level but also within broader healthcare systems.

Despite these promising observations, the optimal use of oritavancin in BJIs remains to be fully defined. Important questions regarding dosing strategies, treatment duration, patient selection, and long-term outcomes persist. Its use should therefore be individualized, guided by clinical judgment, surgical considerations, and microbiological factors, and integrated within a multidisciplinary treatment approach.

In summary, oritavancin represents a practical and potentially impactful alternative to conventional intravenous therapies in selected patients with Gram-positive bone and joint infections. Future prospective studies and controlled trials are essential to establish standardized protocols, refine dosing strategies, and clarify its precise role in the management of these complex infections.

## Figures and Tables

**Table 1:** Summary of the included studies (listed from more recent to older).

Author, Journal, Year	Number of Patients	M/F	Mean Age	Pathogen	Infection Site	Study Design	Intervention	Follow Up	Improvement
Jordan, *Open Forum Infectious Diseases*, 2025 [30]	6 patients (ORI: 6)	52% vs. 48% (whole study)	43 (whole study)	MSSA, PSSA	Osteomyelitis, septic arthritis, fracture fixation device infection	Retrospective single-arm cohort study	ORI 1200 mg single or multiple dose	180 days	ORI 6/6 (100%)
Krsak, *Clinical Orthopaedics and Related research*, 2025 [29]	11 patients (ORI: 6)	54.5% vs. 45.5% (whole study)	55.4 (whole study)	VRE	osteomyelitis, native septic arthritis, prosthetic joint infection	retrospective, multicenter, observational case series	ORI 1200 mg multiple dose weekly or first dose 1200 mg followed by 800 mg weekly	From 7.5 weeks to 3 years and 3 months (treatment Group); from 21 to 48 months (suppressive group)	ORI 2/6 (treatment group, 1 Lost to follow, 1 end of life care) ORI 5/5 (Suppressive Group)
Bandaranayake, *JAC Antimicrob Resist*, 2024 [31]	31 patients (ORI: 31)	62.1% vs. 37.9% (whole study)	Male: 55 Female: 57.5 (whole study)	MRSA, MSSA, CoNS (MRSE), VRE, Group B Streptococci, *C. striatum*, *E. faecalis*, *C. acnes*	Osteomyelitis, HW-associated infection, prosthetic joint infection	Retrospective study	ORI 1200 mg multiple dose weekly or first dose 1200 mg followed by 800 mg weekly (treatment group) 1200 mg monthly/weekly/every 2 weeks (suppressive group)	Not specified	ORI 85% (whole study)
Bongiovanni, *EJCMID*, 2024 [27]	2 patients (ORI: 2)	100% vs. 0%	72.5	MRSA	Osteomyelitis	Single-center, retrospective case series	ORI initial dose 1200 mg, followed by 800 mg every 10 days for a total of 4 doses	15 months	ORI 2/2 (100%)
Buonomo, *ID Cases*, 2024 [40]	1 patient (ORI: 1)	100% vs. 0%	Between 80–89	MRSE	Prosthetic joint infection	Case report	ORI 1200 mg 10 doses in 28 weeks	48 weeks	ORI 1/1 (100%)
Schulz, *Pharmacotherapy*, 2024 [32]	4 patients (ORI: 4)	25% vs. 75%	56.25	MSSA, *Klebsiella oxytoca*, *Citrobacter freundii*	Osteomyelitis, septic arthritis	Retrospective cohort analysis	ORI 1200 mg multiple dose	6 weeks after last dose	ORI 4/4 (100%)
Steuber, *IJAA*, 2024 [33]	124 patients (ORI: 85; DAL: 86)	68% vs. 32% (whole study)	57 (whole study)	MSSA, MRSA, *Streptococcus* spp., CoNS, *Enterococcus faecalis*	Osteomyelitis, prosthetic joint infection, diabetic foot	Single-centre, retrospective, case–control study	ORI 1200 mg every 7–14 days followed by 800 mg every 7–14 days vs. DAL 1000 mg or 1500 mg every 7–14 days	Not specified	For osteomyelitis, ORI: 48/59 (81%); DAL 48/65 (74%)
Van Hise, *Infect Dis Ther*, 2024 [34]	150 patients (ORI: 75; DPT: 75)	51% vs. 49%	64.6 ± 13.4	MSSA, MRSA, VRE, other Gram-positive spp.	Osteomyelitis	Retrospective, observational study	ORI initial dose 1200 mg, followed by 800 mg on day 8 of treatment vs. DPT 6–8 mg/kg for 4–6 weeks	7–10 days after, 3 months after, 6 months after last dose	ORI 55/75 (73.3%) DPT 25/75 (33.4%)
Ahiskali, *BMC Pharmacology and Toxicology*, 2020 [35]	14 patients (ORI: 14)	71.4% vs. 28.6%	Not specified	MSSA, MRSA, MRSE, *Streptococcus mitis/oralis*	Osteomyelitis, joint infection	Retrospective chart review	ORI 1200 mg single or multiple dose	Up to 60 days after final infusion	ORI 10/11 (90.9%)
Brownell, *Drugs-Real World Outcomes, 2020* [28]	16 patients (ORI: 16)	50% vs. 50%	50 (whole study)	Not specified	Osteomyelitis, prosthetic joint infection, diabetic foot	Single-center, retrospective case series	ORI initial 1200 mg dose followed by 1200 mg or 800 mg weekly	Not specified	ORI: 16/16 (100%)
Nguyen, *ID Cases*, 2020 [41]	1 patient (ORI: 1)	0% vs. 100%	62	VRE	Prosthetic joint infection	Case report	ORI 1200 mg weekly for 6 weeks	10 months after last dose	ORI 1/1 (100%)
Van Hise, *Drugs-Real World Outcomes*, 2020 [36]	130 patients (ORI: 130)	49.3% vs. 50.7%	60 (19–97)	MSSA, MRSA, VRE, VISA	Osteomyelitis	Multicenter, retrospective, descriptive study	ORI initial dose 1200 mg, then 800 mg weekly for a total of four (n = 118) or five (n = 16) weeks	7–10 days after, 3 months after, 6 months after last dose	ORI 104/130 (80%)
Chastain, *IJAA*, 2019 [37]	9 patients (ORI: 9)	66.3% vs. 33.3%	65 (median)	MRSA	Osteomyelitis	Retrospective chart review	ORI 1200 mg weekly	6 months	ORI 9/9 (100%)
Dahesh, *Antimicrob Agents Chemoter*, 2019 [42]	1 patient (ORI + AMP: 1)	100% vs. 0%	59	VRE	HW-associated infection	Case report	ORI 1200 mg weekly for 2 weeks followed by 800 mg weekly for 8 weeks + AMP 12 g per day as continuous infusion	10 weeks	ORI + AMP 1/1 (100%)
Redell, *OFID*, 2019 [49]	28 patients (ORI: 28)	53.2% vs. 46.8% (whole study)	57.8 (whole study)	MSSA, MRSA, *Streptococci* spp.	Osteomyelitis, prosthetic joint infection, septic arthritis or synovitis, infected bursa	Retrospective observational program	ORI 1200 mg single or multiple dose	Not specified	ORI 10/11 (90.9%) for multiple-dose administration
Ruggero, *Infect Dis Clin Pract*, 2018 [39]	1 patient (ORI: 1)	100% vs. 0%	46	MRSA	Osteomyelitis	Case report	ORI 1200 mg every 2 weeks for 8 weeks	1 year	ORI 1/1 (100%)
Delaportas, *Pharmacotherapy*, 2017 [43]	1 patient (ORI: 1)	0% vs. 100%	49	MSSA	HW-associated infection	Case report	ORI 1200 mg weekly for 6 weeks	40 weeks after last dose	ORI 1/1 (100%)
Stewart, *Infectious Diseases and Therapy*, 2017 [21]	1 patient (ORI: 1)	0% vs. 100%	26	MSSA	Osteomyelitis	Case report	ORI 1200 mg single dose	6 weeks	Not specified

Abbreviations: ORI—oritavancin; DPT—daptomycin; DAL—dalbavancin; AMP—ampicillin; MSSA—methicillin-sensitive *Staphylococcus aureus*; MRSA—methicillin-resistant *Staphylococcus aureus*; MRSE—methicillin-resistant *Staphylococcus epidermidis*; PSSA—penicillin-sensitive *Staphylococcus aureus*; VISA—vancomycin-intermediate *Staphylococcus aureus*; VRE—vancomycin-resistant Enterococci; CoNS—coagulase-negative Staphylococci; HW-associated infection—hardware-associated infection.

## Data Availability

No new data were generated or analyzed in this study.
